# QuickStats

**Published:** 2014-01-10

**Authors:** FROM THE NATIONAL CENTER FOR HEALTH STATISTICS

**Figure:**
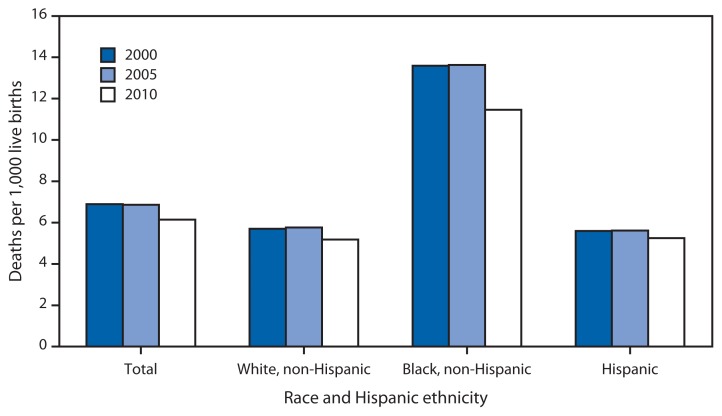
Infant Mortality Rates,* by Race and Hispanic Ethnicity of Mother — United States, 2000, 2005, and 2010 * Per 1,000 live births.

The U.S. infant mortality rate plateaued during 2000–2005, then declined from 6.86 infant deaths per 1,000 live births in 2005 to 6.14 in 2010. Declines from 2005 to 2010 were largest for non-Hispanic black women (from 13.63 to 11.46), followed by non-Hispanic white (from 5.76 to 5.18) and Hispanic women (from 5.62 to 5.25). In 2000 and 2005, the non-Hispanic black infant mortality rates were 2.4 times the non-Hispanic white rates; however, the difference between the two rates has narrowed, and in 2010, the non-Hispanic black rate was 2.2 times the non-Hispanic white rate.

**Source:** Mathews TJ, MacDorman MF. Infant mortality statistics from the 2010 period linked birth/infant death data set. Natl Vital Stat Rep 2013;62(8).

**Reported by:** Marian F. MacDorman, PhD, mfm1@cdc.gov, 301-458-4356; T.J. Mathews.

